# Evolutionary History of Hunter-Gatherer Marriage Practices

**DOI:** 10.1371/journal.pone.0019066

**Published:** 2011-04-27

**Authors:** Robert S. Walker, Kim R. Hill, Mark V. Flinn, Ryan M. Ellsworth

**Affiliations:** 1 Department of Anthropology, University of Missouri, Columbia, Missouri, United States of America; 2 School of Human Evolution and Social Change, Arizona State University, Tempe, Arizona, United States of America; Virginia Tech Virginia, United States of America

## Abstract

**Background:**

The universality of marriage in human societies around the world suggests a deep evolutionary history of institutionalized pair-bonding that stems back at least to early modern humans. However, marriage practices vary considerably from culture to culture, ranging from strict prescriptions and arranged marriages in some societies to mostly unregulated courtship in others, presence to absence of brideservice and brideprice, and polyandrous to polygynous unions. The ancestral state of early human marriage is not well known given the lack of conclusive archaeological evidence.

**Methodology:**

Comparative phylogenetic analyses using data from contemporary hunter-gatherers around the world may allow for the reconstruction of ancestral human cultural traits. We attempt to reconstruct ancestral marriage practices using hunter-gatherer phylogenies based on mitochondrial DNA sequences.

**Results:**

Arranged marriages are inferred to go back at least to first modern human migrations out of Africa. Reconstructions are equivocal on whether or not earlier human marriages were arranged because several African hunter-gatherers have courtship marriages. Phylogenetic reconstructions suggest that marriages in early ancestral human societies probably had low levels of polygyny (low reproductive skew) and reciprocal exchanges between the families of marital partners (i.e., brideservice or brideprice).

**Discussion:**

Phylogenetic results suggest a deep history of regulated exchange of mates and resources among lineages that enhanced the complexity of human meta-group social structure with coalitions and alliances spanning across multiple residential communities.

## Introduction

Marriage is a human universal that unites males and females in socially-recognized reproductive units [1]. While stable-breeding bonds are found in numerous taxa, human marriages have wide significance beyond reproduction. Marriage is a fundamental cornerstone of human economic, social, and kinship networks [1]–[6]. Indeed, marriage as an elementary principle of human kinship systems has long been considered a central aspect of between-group alliances [5]. The exchange of mates among kin groups (reciprocal exogamy) and accompanying networks of economic exchange (e.g., brideservice and brideprice) are widespread and arguably create the foundation of human social organization [2], [3]. However, considerable cultural variation around the world opens up the question of whether regulated exchange of mates across kin groups represents the ancestral form of marriage or whether it is a recently derived consequence of more intensive modes of subsistence. This question is important to answer because in some societies marriage is a nonchalant affair with limited regulation in courtship marriages with no prescriptions (although proscriptions against close kin are ubiquitous), while in others marriages are arranged and regulated by complex rules and prescriptions [2], [7]. To address these questions about the evolutionary history of human marriage, we present phylogenetic reconstructions of marriage practices using comparative data from contemporary hunter-gatherers.

Humans lived as hunter-gatherers for most of our species' history hence cultural variation amongst recent hunter-gatherers may be useful for reconstructing ancestral human social structure [8]–[10]. In a comparative study of 190 hunter-gatherer societies, Apostolou [11] showed that arrangement of marriage by parents or close kin is the primary mode of marriage in 85% of the sample; brideservice, brideprice, or some type of exchange between families is found in 80% of the sample; and less than 20% of men are married polygynously in 87% of the sample. The prevalence of marriage practices in hunter-gatherers suggests a deep history of regulated marriage. Brideservice and brideprice are often crucial economic components of regulated mate exchange, and low levels of polygyny indicate evenness of such exchanges. Here we further investigate marriage variation by adding time-depth via phylogenetic analyses in order to better formulate evolutionary sequences of derived human traits surrounding marriage (e.g., mate exchange, meta-group social structure, etc.).

The phylogenetic reconstruction of marriage practices is important for several reasons. First, phylogenetic reconstructions permit insight into the ancestral state of human marriage and track the cultural inertia of particular types of marriage practices. Second, the evolutionary history of critical components of human marriage practices aids our ability to formulate informed hypotheses about the evolution of variation in human social structure. Finally, some evolutionary researchers have stressed the importance of autonomous mate choice in shaping human behavior and morphology [12], [13], while others [14]–[16] have been more cautious about the extent to which individuals are able to freely choose mates, given that mating and marriage is often heavily regulated by parents and close kin [11]. Phylogenetic analyses yield evidence on the extent to which ancestral males and females were able to realize their own mate preferences and thus the nature of human sexual selection.

## Results

**Figure 1:**
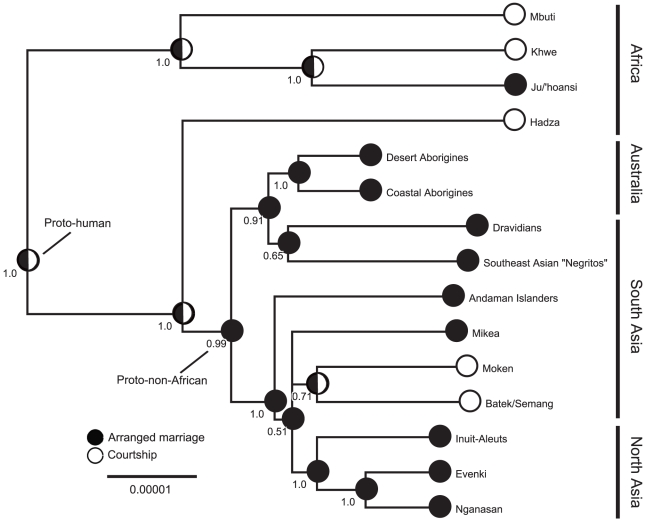
Majority-rule consensus tree of 1,000 MCMC phylogenies using mitochondrial DNA sequences from modern hunter-gatherers. Node numbers represent posterior probability support for particular clades. Arranged marriages (black) versus courtship practices (white) are reconstructed across 1,000 trees with stochastic character mapping (Bayesian analysis). Node circles represent ancestral reconstructions for marriage practices where half-black/half-white circles represent equivocal reconstructions. Plural taxa names represent multiple representative cultures (see Table 2).

As expected from previous genetic studies, the mitochondrial DNA phylogenies generated here (Figure 1) show the deepest divergences in Africa, first by San speakers (Ju/'hoansi, or !Kung San, and Khwe) and African “Pygmies”, followed by the Hadza of Tanzania. The next clade to split off includes Australian Aborigines, hunter-gatherers on the Indian subcontinent (Dravidian language family), and the “Negritos” of Southeast Asia. The final clade to diverge includes several northern latitude hunter-gatherers (Inuit-Aleut, Nganasan, and Evenki). These phylogenetic trees are generally congruent with more extensive studies of global genetic variation [17]–[19].

The reconstruction of human marriage practices for ancestral humans show several consistent patterns using Bayesian, maximum likelihood, and parsimony methods (Table 1). The reconstruction of low levels of polygyny in early humans is straightforward because high levels of polygyny for hunter-gatherers are only found in Australian Aborigines and are mostly low elsewhere (most exceptions are some New World foragers that are not in the phylogenetic analysis). Low levels of polygyny and low reproductive skew among ancestral humans are consistent with human morphology and behavior (i.e., moderate sperm counts [20] and testicular size [21]; facultative paternal investment [22]) and the general decline in sexual dimorphism beginning at least with early *Homo* [23].

**Table 1:** Likely ancestral states for proto-human and proto-non-African cultures using 3 different reconstruction methods.

	Bayesian		Maximum Likelihood		Maximum Parsimony	
	Proto-	Proto-	Proto-	Proto-	Proto-	Proto-
Cultural trait	human	non-African	human	non-African	human	non-African
Arranged marriage	equivocal	yes (0.99)	equivocal	yes (0.97)	no (0.99)	yes (0.97)
Brideservice/price	yes (1.0)	yes (1.0)	yes (0.99)	yes (1.0)	yes (1.0)	yes (0.99)
Polygyny prevalence	low (1.0)	low (0.99)	low (1.0)	low (0.99)	low (1.0)	low (0.99)

Numbers in parentheses represent the proportion of 1,000 MCMC trees that support the reconstruction.

The existence of brideprice, brideservice, or both is the most likely ancestral state for humans according to all 3 reconstruction methodologies. Some type of exchange of goods or labor between the families of marital partners, not including token brideprice, is found in 80% of Apostolou's [11] full sample. Brideprice/service is recorded for most hunter-gatherers in the reduced sample with the exception of the Mikea (brideprice is token only), Batek, and Andaman Islanders (Table 2). Given that brideservice and brideprice are often crucial economic components of regulated mate exchange, a deep history of these practices may in and of itself indicate a deep history of regulated marriage.

**Table 2:** Hunter-gatherer marriage data.

			GenBank	HvrBase		Brideservice/	Polygyny
Culture(s)	Representative culture(s)	Region	ascension	Id	Marriage	brideprice	prevalence
Mbuti	Mbuti, Efe, “Pygmies”	Africa	-	15236	courtship	yes	low
Khwe	G/wi	Africa	-	15909	courtship	yes	low
Ju/'hoansi	Ju/'hoansi (!Kung)	Africa	EF184590	-	arranged	yes	low
Hadza	Hadza	Africa	EF184619	-	courtship	yes	low
Desert Aborigines	Yolgnu, Walbiri, Arrente, Pintupi	Australia	DQ404441	-	arranged	yes	high
Coastal Aborigines	Gidjingali, Gunwinggu	Australia	DQ404440	-	arranged	yes	high
Dravidians	Chenchu, Paliyan, Kadar	India	FJ467950	-	arranged	yes	low
SE Asian “Negritos”	Batak, Agta, Aeta	SE Asia	GU733756	-	arranged	yes	low
Andaman Islanders	Onge, Andamanese, Jarawa	Andamans	DQ149517	-	arranged	no	low
Mikea	Mikea	Madagascar	FJ543101	-	arranged	token	low
Moken	Moken	Myanmar	FJ442938	-	courtship	yes	low
Semang/Batek	Batek De, Semang	Malaysia	AY963576	-	courtship	no	low
Aleut-Inuits	Inuit, Yupik, Aleut	Alaska	-	3818	arranged	yes	low
Evenki	Evenki	Russia	-	15222	arranged	yes	low
Nganasan	Nganasan	Siberia	-	3473	arranged	yes	low

The evolution of courtship versus arranged marriages in early humans is more difficult to reconstruct. Because 3 of 4 African hunter-gatherers in the phylogeny are coded as having courtship marriages, maximum parsimony reconstructs the ancestral proto-human root as having courtship and makes the maximum likelihood and Bayesian reconstructions equivocal (Figure 1). Put simply we do not yet know whether or not mitochondrial Eve's marriage was arranged. In Apostolou's [11] full sample, 3 of 8 African hunter-gatherers have courtship marriages (all 3 are included in the phylogenetic analysis). This may imply that African hunter-gatherers with courtship have switched from arranged to courtship marriages since the last common ancestor, perhaps under pressure from recent Bantu expansions. All 3 reconstruction methods support arranged marriages for proto-out-of-Africa (proto-non-African). Therefore, regardless of the ancestral state of early humans, arranged marriages probably have an evolutionary history going back at least 50,000 years.

In the full sample there is a statistical relationship between arranged marriage and presence of brideprice or brideservice (Pearson Chi-square = 9.456, *df* = 1, *p* = 0.014, *n* = 185) and between types of arranged marriage and the prevalence of polygyny (Pearson Chi-square = 13.204, *df* = 2, *p* = 0.001, *n* = 76) with more polygyny when kin, but not parents, arrange marriages. Marlowe [10] also reports a relationship between higher percentage of polygynous women and arrangement of marriages for females. Arranged marriage is not related to socio-environmental variables from Binford's comparative hunter-gatherer database [24], such as latitude, temperature, habitat, mobility, dietary quality, population density, or net primary productivity. None of these variables reached a significance level (*p*-value) less than 0.05 even though all have sample sizes of at least 96 hunter-gatherer societies. If marriage arrangement practices adapted relatively quickly to local environmental situations, we might expect a correlation with one or more of these variables. In sum, the arrangement of marriage in hunter-gatherers is not easily predicted by environmental context but does co-vary with brideservice/price and with more polygyny and larger families when kin arrange marriages.

## Discussion

Our phylogenetic results support a deep evolutionary history of limited polygyny and brideprice/service that stems back to early modern humans and, in the case of arranged marriage, to at least the early migrations of modern humans out of Africa. It is conceivable that marriage involved some level of arrangement, regulation, and reciprocal relationships from the very earliest inception of marriage-like cultural institutions. The presence of brideprice or brideservice as the ancestral human state may be interpreted as early critical components of regulated mate exchange. The very act of a male moving away from his kin and community (e.g., brideservice) is a tremendous leap from the insular patterns in other apes. It is an indication of negotiation between kin groups and the recognition of a continued set of obligations and reciprocal transactions (alliance) between the families. This, combined with the low prevalence of polygyny as the ancestral human state, suggests that there was a reasonable level of evenness to mate exchanges (low reproductive skew).

Marriage practices may be expected to be labile traits changing rapidly with ecological conditions, but our reconstructions actually suggest that these traits may change slowly over time, at least for hunter-gatherers in the absence of pressure from neighboring agriculturalists. Case in point is Australia where Aborigines across the continent heavily regulated marriage probably over many millennia and had no traditional exposure to agriculture. Conservatism in marriage practices, which is the justification for using phylogenetic methods in the first place, is tentatively supported by the universality of marriage around the world, the 85% prevalence of arranged marriages across hunter-gatherer societies, and the lack of relationship between arrangement of marriage and environmental variables. Social exigencies driven by the importance of alliances/coalitions and norms of reciprocity are what have likely consistently favored the regulation of marriage and mate exchange networks in human societies over considerable time and space. The cultural phylogenies presented here are probably not driven simply by high-fidelity transmission of arbitrary marriage practices in some type of blind process of copying previous generations. Instead, common marriage practices are likely adaptations to common social circumstances of hunter-gatherers demonstrating deep evolutionary roots of core human cultural traits.

Modern hunter-gatherers are not Pleistocene relics and may not be direct, unbroken descendants of ancestral hunter-gatherers. Marginal habitats, pressure from agricultural neighbors, and assimilation and acculturation into state-level societies have all significantly affected hunter-gatherer lifeways. However, these processes have most likely served to disrupt traditional cultural norms [25] in ways that simplify or de-regulate marriage practices, as opposed to strengthening marriage regulation. We suspect that early human marriages were regulated and that hunter-gatherers lacking regulation of marriage (e.g., several African hunter-gatherers), brideprice/service, and other social complexities, may have recently lost these traits in the face of contact with more powerful neighbors.

Our reconstruction of the evolutionary history of hunter-gatherer marriage practices indicates that parents and other close kin likely had a significant influence on mate choice. How regulated marriage affected sexual selection on human mate choice preferences depends on several factors. One factor is the extent to which parental (and other senior kin) choices overlapped or diverged with that of offspring mate choice. Another factor is the extent to which marital partners chosen by parents were the actual genitors of the descendants. Worldwide extra-pair paternity rates have been estimated at around 9% [26], although there is much variation between as well as within populations [27]. Regarding the first issue, parent-offspring conflict over mate choice in contemporary Western populations has been found to contain considerable conflict of interest, as well as some expected overlap, in preferred attributes [28]–[31]. However, environmental novelty may render these findings unrepresentative of ancestral situations and a systematic examination of parental and offspring mate choice preferences among hunter-gatherers and other small-scale societies is warranted. At present, it is probably safe to conclude that an important selective pressure on the evolution of human mate choice, certainly more than any other species, has been the direct, deliberate, and conscious intervention of parents and other close kin on the sexual lives of their descendants.

**Figure 2:**
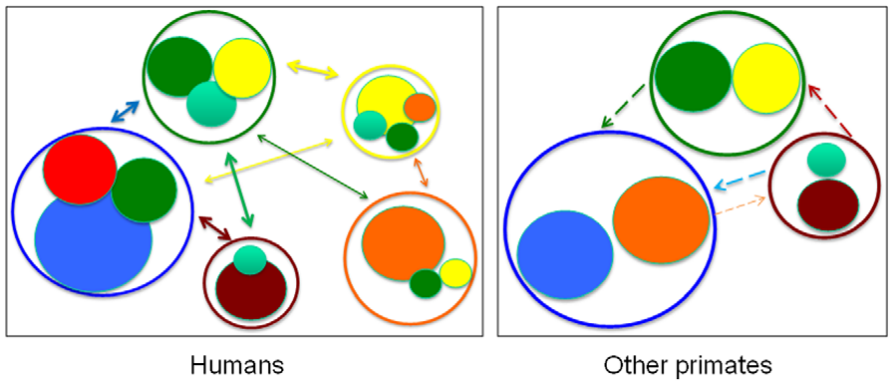
The evolved human social structure (left) of reciprocal exogamy including the exchange of mates, goods, and services (double-headed arrows), involves multiple kin lineages (filled circles) often existing in multiple residential communities (open circles). Extensive cooperation (overlap of filled circles) likely results in economies of scale within and across human communities. In contrast, in other primates (right) one or the other sex emigrates (dotted arrows). The lack of any reciprocal exogamy means that kin lineages are isolated to single communities and thus do not generate a meta-group social structure as found in humans [2], [3].

Humans evolved a novel social structure with regulated marriage and reciprocal mate exchange (Figure 2). Chapais [2], [3] has showed how stable breeding bonds, paternity recognition, and between-group transfer paved the way for amicable relations between groups and the evolution of a meta-group social structure via bilateral kin and in-law recognition spanning across human communities. The regulation of marriage practices probably evolved later, further establishing more complex between-group reciprocal alliances, and creating the variety of meta-group social dynamics characteristic of modern humans. Most cultures have prescriptions (and all have proscriptions) that specify what categories of kin are appropriate (or not) for mating and marriage and have rules and preferences concerning resulting kin ties [32]. Marriage prescriptions commonly involve real or classificatory cross-cousins and naturally emerge as a result of multiple generations of sister or daughter exchange between two kin lineages [2], [5], [7]. Complex networks of human reciprocal exogamy, often including critical economic components of brideservice and brideprice, frequently involve kin groups existing in multiple residential communities. A key resulting feature of the evolved human social system is the affiliation of several unrelated males to the same female (e.g., related as wife, daughter, sister, or brother's wife) that may ameliorate hostile relations across patrilineages and facilitate opportunistic visiting and co-residence [2], [3], [33], [34]. In contrast, other primates lack reciprocal exogamy and kin lineages are isolated to single communities [35]. Therefore, unlike other primates, human cooperation transcends local residential units and mutualistic or reciprocal relationships with neighboring bands emerge via the recognition of bilateral kinship relationships across multiple communities [2], [3]. The causes and consequences of this evolved human social structure, and the full nature of its norms and regulation, remain fascinating areas for future research.

## Methods

To generate hunter-gatherer phylogenies, the websites HvrBase++ and GenBank were queried for complete mitochondrial DNA (mtDNA) sequences from well-studied hunter-gatherer societies where information is available on arranged versus courtship marriages, presence versus absence of brideprice and brideservice, and prevalence of polygyny (coded as low if percent of men married polygynously <20%, or high otherwise). Marriage data are from Apostolou's [11] comparative hunter-gatherer study. There are 15 matches between cultural and genetic datasets; 7 of these matches represent cultural areas (e.g., Inuit-Aleuts) that include multiple representative cultures homogenous for particular marriage practices (Table 2). The distribution of traits for the 15 matches is approximately the same as in Apostolou's [11] full sample (i.e., 67% arranged versus 85% arranged in full sample; 80% brideservice/price in both full and reduced samples; 87% with low polygny versus 78% in full sample). The Mikea hunter-gatherers of Madagascar included here were likely agriculturalists in the recent past (respecialized foragers) but their omission has no effect on results. Horticultural, agricultural, and pastoral societies were not included because they represent recent economic adaptations. Including agropastoralists would bias the sample towards even more regulated marriage systems [36]. New World hunter-gatherers other than Inuit-Aleuts were not included because they integrated phylogenetically with North Asian hunter-gatherers in ways that misrepresented the colonization of the New World. Mitochondrial DNA variation is ideally suited towards resolving deep phylogenetic relationships greater than 10,000 years or so [37] but is not as valid for more recent relationships for which linguistic variation is more appropriate [38].

### Tree building

Mitochondrial sequences were manually aligned with the revised Cambridge Reference Sequence [39]. Aligned sequences were then used to construct phylogenies using a general time-reversible model with gamma-distributed rate variation (GTR+Γ+I) and a strict molecular clock in BEAST version 1.6.1 [40]. BEAST does not require an out-group to be specified but instead samples the root position along with the rest of the nodes in the tree. Posterior distributions of parameters were estimated by Markov chain Monte Carlo (MCMC) sampling. Samples were drawn every 10,000 MCMC generations from a total of 20,000,000 generations. The initial half of runs was disregarded to allow for ample burn-in. Tracer software was used to verify convergence to a stationary distribution and sufficient sampling. This method yielded 1,000 hunter-gatherer phylogenies used for the reconstruction of marriage traits.

### Marriage phylogenetics

Marriage data (arranged versus courtship, presence versus absence of brideservice or brideprice, and low versus high levels of polygyny) were reconstructed by mapping variation onto mtDNA phylogenies. Three separate reconstruction methods were used in Mesquite software [41]: 1) Bayesian stochastic character mapping [42], 2) maximum likelihood, and 3) maximum parsimony. All three methods account for phylogenetic uncertainty by running reconstructions over the posterior sample of 1,000 MCMC trees. The advantage of the Bayesian and maximum likelihood techniques is that they explicitly model the rate of loss and gain of particular traits [43]. In contrast, maximum parsimony simply minimizes the total number of losses and gains. Ancestral reconstruction using maximum parsimony is driven by the states of the earliest branch to diverge (in this case, African hunter-gatherers).

### Marriage correlates

Chi-square tests were run to examine statistical relationships between arranged marriage, polygyny prevalence, and brideprice or brideservice in SPSS (version 17.0). Multinomial regression tests of arranged marriage using 3 levels (courtship, parents arrange, kin arrange) were regressed on socio-environmental variables from Binford's comparative hunter-gatherer database [24]. These variables include latitude, effective temperature, mean temperature, ecology (tropical forest, boreal forest, desert, grassland/shrubland, or polar tundra), mobility (total distance moved per year), dietary quality, net primary productivity, and population density at the ethnolinguistic level.
